# OA15 Refractory Takayasu arteritis: challenges in disease monitoring and therapeutics - a case series

**DOI:** 10.1093/rap/rkag113.015

**Published:** 2026-11-03

**Authors:** Reshma Seomore, Emma Davies, Bashaar Boyce, Elizabeth Reilly, Elizabeth Perry, Robert Marshall, Matthew Roy, Keziah Austin, Andreia Ferreira, Daria Walewska, Joanna Robson, Kian Wah Lim

**Affiliations:** Department of Rheumatology, Bristol NHS Foundation Trust, Bristol, United Kingdom; Department of Rheumatology, Bristol NHS Foundation Trust, Bristol, United Kingdom; Department of Rheumatology, Bristol NHS Foundation Trust, Bristol, United Kingdom; Department of Rheumatology, Bristol NHS Foundation Trust, Bristol, United Kingdom; Department of Rheumatology, Bristol NHS Foundation Trust, Bristol, United Kingdom; Department of Rheumatology, Bristol NHS Foundation Trust, Bristol, United Kingdom; Department of Rheumatology, Bristol NHS Foundation Trust, Bristol, United Kingdom; Department of Rheumatology, Bristol NHS Foundation Trust, Bristol, United Kingdom; Department of Rheumatology, Bristol NHS Foundation Trust, Bristol, United Kingdom; Department of Rheumatology, Bristol NHS Foundation Trust, Bristol, United Kingdom; Department of Rheumatology, Bristol NHS Foundation Trust, Bristol, United Kingdom; Department of Rheumatology, Bristol NHS Foundation Trust, Bristol, United Kingdom

## Abstract

**Introduction:**

Takayasu arteritis (TAK) is a chronic granulomatous large-vessel vasculitis affecting the aorta and its primary branches. Assessment of disease activity remains challenging, particularly during interleukin-6 (IL-6) inhibition, where inflammatory markers may not reliably reflect ongoing vascular inflammation. We present two cases of refractory TAK in which persistent disease activity during tocilizumab therapy required integration of clinical assessment and multimodal vascular imaging to guide escalation to infliximab.

**Case description:**

Case1: A 27-year-old woman, presented with abdominal pain, episcleritis and elevated inflammatory markers (peak CRP 263 mg/L). CT thorax and PET-CT demonstrated active supradiaphragmatic large-vessel vasculitis involving the aorta and proximal head and neck vessels, while carotid Doppler ultrasound showed circumferential arterial inflammation with marked bilateral common carotid narrowing. She received glucocorticoids and methotrexate but relapsed with carotidynia, upper-limb claudication, headache and visual symptoms. Tocilizumab was introduced and switched from subcutaneous to intravenous therapy, while methotrexate was replaced with mycophenolate mofetil (MMF) because of deranged liver function tests. Despite normal inflammatory markers during tocilizumab therapy, serial imaging demonstrated persistent vascular inflammation, with worsening FDG uptake on PET-CT. Following multidisciplinary review, tocilizumab was switched to infliximab because of persistent disease activity and associated inflammatory arthritis. Follow-up PET-CT showed only mild residual FDG uptake.

Case2: A 30-year-old woman with Takayasu arteritis had extensive large-vessel involvement disease affecting the aorta branch vessels. Following glucocorticoid withdrawal and MMF reduction, she relapsed with recurrent left upper-limb pain, neck pain, headache and abdominal symptoms. CT angiography and carotid Doppler ultrasound demonstrated persistent large-vessel disease, whereas PET-CT was repeatedly limited by marked brown-fat activation. Inflammatory markers remained normal during this and subsequent relapses despite ongoing clinical and radiological disease activity. MMF escalation and reintroduction of tocilizumab resulted in an initial clinical response. Following glucocorticoid cessation, she relapsed with left supraclavicular swelling, left upper-limb pain and pleuritic chest pain. CT angiography demonstrated progressive left common carotid narrowing despite normal inflammatory markers and non-diagnostic PET-CT. Glucocorticoids were restarted and tocilizumab was switched to intravenous therapy. Despite this, she relapsed with chest pain, breathlessness, inflammatory arthritis and persistent left supraclavicular swelling. CT angiography and gated thoracic CT confirmed active Takayasu arteritis, indicating secondary tocilizumab failure. Tocilizumab was switched to infliximab, with early symptomatic improvement, although left supraclavicular swelling persisted.

**Discussion:**

These cases illustrate the challenges of assessing disease activity and treatment response in refractory TAK during IL-6 blockade. In both patients, recurrent vascular symptoms and serial multimodal imaging demonstrated ongoing active disease activity despite normal inflammatory markers. Assessing disease activity using CRP is not reliable during IL-6 blockade, which adds to the challenges of monitoring and ongoing management.

The first case demonstrated persistent vascular inflammation and worsening FDG uptake on serial PET-CT despite a switch from subcutaneous to intravenous tocilizumab. The second case further illustrated the challenges and limitations of disease monitoring, with repeated PET-CT assessments rendered difficult by marked brown-fat activation despite progressive vascular abnormalities on CT angiography and Doppler ultrasound. These cases show the importance of integrating clinical assessment with complementary imaging modalities when disease activity remains uncertain.

The 2021 American College of Rheumatology/Vasculitis Foundation guideline conditionally recommends TNF inhibitors over tocilizumab in glucocorticoid-refractory TAK, while recent EULAR recommendations support consideration of both IL-6 inhibition and TNF inhibition in refractory TAK. In both cases, serial imaging demonstrated persistent or progressive vascular disease despite glucocorticoids, conventional immunosuppressive therapy and tocilizumab. Following multidisciplinary review, infliximab was selected because of ongoing refractory disease, including an inflammatory arthritis element of disease activity.

In the UK, tocilizumab has traditionally been the only commissioned biologic for refractory TAK. Evolving access to infliximab may provide an additional treatment option for patients with refractory disease, as illustrated by these cases.

**Key learning points:**

1. Normal inflammatory markers do not exclude active Takayasu arteritis during IL-6 blockade. In both cases, ongoing vascular inflammation was demonstrated radiologically despite normal and falsely reassuring inflammatory markers, emphasising the limitations of biomarkers monitoring.

2. Assessment of disease activity required integration of serial CT angiography, carotid Doppler ultrasound, MRA and PET-CT. These modalities provided complementary information, with CT angiography and carotid Doppler ultrasound proving particularly valuable when inflammatory markers were normal and PET-CT findings were difficult to interpret or discordant with the clinical picture.

3. PET-CT has its limitations. In our second patient, its utility was limited by marked brown fat uptake, emphasising the need for correlation with clinical findings and consideration of alternative imaging modalities.

4. In refractory Takayasu arteritis despite tocilizumab, treatment escalation should be guided by clinical assessment, serial imaging and multidisciplinary review. In both cases, persistent vascular disease prompted switching of therapy, with an inflammatory arthritis element contributing to the overall assessment of refractory disease.

